# Role of PARP Inhibitors in Glioblastoma and Perceiving Challenges as Well as Strategies for Successful Clinical Development

**DOI:** 10.3389/fphar.2022.939570

**Published:** 2022-07-06

**Authors:** Priya Bisht, V. Udaya Kumar, Ruchi Pandey, Ravichandiran Velayutham, Nitesh Kumar

**Affiliations:** ^1^ Department of Pharmacology and Toxicology, National Institute of Pharmaceutical Education and Research (NIPER-Hajipur), Hajipur, India; ^2^ Department of Pharmacy Practice, National Institute of Pharmaceutical Education and Research (NIPER-Hajipur), Hajipur, India

**Keywords:** GBM, TMZ, PARP inhibitors, PARP inhibitor resistance, hematological toxicity, targeted delivery, combination therapy

## Abstract

Glioblastoma multiform is the most aggressive primary type of brain tumor, representing 54% of all gliomas. The average life span for glioblastoma multiform is around 14–15 months instead of treatment. The current treatment for glioblastoma multiform includes surgical removal of the tumor followed by radiation therapy and temozolomide chemotherapy for 6.5 months, followed by another 6 months of maintenance therapy with temozolomide chemotherapy (5 days every month). However, resistance to temozolomide is frequently one of the limiting factors in effective treatment. Poly (ADP-ribose) polymerase (PARP) inhibitors have recently been investigated as sensitizing drugs to enhance temozolomide potency. However, clinical use of PARP inhibitors in glioblastoma multiform is difficult due to a number of factors such as limited blood–brain barrier penetration of PARP inhibitors, inducing resistance due to frequent use of PARP inhibitors, and overlapping hematologic toxicities of PARP inhibitors when co-administered with glioblastoma multiform standard treatment (radiation therapy and temozolomide). This review elucidates the role of PARP inhibitors in temozolomide resistance, multiple factors that make development of these PARP inhibitor drugs challenging, and the strategies such as the development of targeted drug therapies and combination therapy to combat the resistance of PARP inhibitors that can be adopted to overcome these challenges.

## 1 Introduction

Glioma is defined as one of the primary brain tumors that are categorized based on their origin cells such as astrocytic tumors (anaplastic astrocytoma or glioblastoma multiform), oligodendrogliomas, ependymomas, and mixed gliomas. They are among the most prevalent types of tumors present in the central nervous system (CNS), accounting for over 80% of overall primary brain tumors which are malignant (Hanif et al., 2017). Glioblastoma multiform (GBM)/glioblastoma is one such type of glioma which is highly prevalent and accounts for 54% of all gliomas, among which malignant primary brain as well as CNS tumors comprise 45.2%, while 16% is carried by primary brain as well as CNS tumors. Additionally, it is an aggressive primary malignant brain tumor, which has been categorized as grade IV type by the World Health Organization (WHO). The average life span for GBM is around 14–15 months after diagnosis (Louis et al., 2007; Ghosh et al., 2018). GBM has a global incidence of 0.59–3.69 per 100,000 live births, with a male-adjusted incidence rate of 3.97 cases per 100,000 and a female-adjusted incidence rate of 2.53 cases per 100,000 (Ghosh et al., 2018). GBM most frequently occurs in the hemispheres of the cerebrum, among which 95% of GBM originates in the supratentorial area, although hardly a small percentage of GBM arises in the cerebellum, brainstem, or spinal cord (Nakada et al., 2011). GBM is divided into two main subtypes: primary and secondary, and the term was first coined in Antwerp in 1940 by the German neuropathologist Hans Joachim Sherer (Kleihues and Ohgaki, 1999). Primary GBM is the most common subtype with 80% of cases and manifests later in life at the median age of 62 years, while secondary GBM manifests earlier in life at the median age of 45 years (Kleihues and Ohgaki, 1999; Ohgaki and Kleihues, 2009; Ohgaki et al., 2004; Watanabe et al., 2009). Primary GBM is associated with overexpression of epidermal growth factor receptor (EGFR) as well as mouse double minute 2 (MDM2) gene along with deletion of p16, loss of heterozygosity (LOH) of chromosome 10q holding PTEN (phosphatase and tensin homolog), and telomerase reverse transcriptase (TERT) promoter mutation while secondary class of GBM originates from low-grade astrocytoma or oligodendrogliomas and often contain mutations in tumor protein 53 (TP53), ATRX, and isocitrate dehydrogenase 1/2 (IDH1/2) or overexpression of LOH of 19q, retinoblastoma (RB), platelet-derived growth factor A, and platelet-derived growth receptor alpha (PDGFA/PDGFRa) (Davis, 2016; Hanif et al., 2017). A rare subtype of GBM named GBM-0 is also added by WHO with an oligodendroglioma feature, which is characterized as GBM with regions resembling anaplastic oligodendroglioma, having GBM-like characteristics and necrosis but without microvascular proliferation (Louis et al., 2007). The current therapy available for the treatment of GBM includes clinical removal of the tumor adjuvant to radiation therapy (RT) with temozolomide (TMZ) administration. TMZ is a first-line chemotherapeutic agent widely used in the treatment of GBM; however, resistance to TMZ is frequently the limiting factor in effective treatment (Gupta et al., 2019; Singh et al., 2021). The principal pathway involved in the induction of resistance toward TMZ is through the activation of multiple DNA repair pathways (MGMT-O^6^-methylguanine DNA methyltransferase, MMR-mismatch repair, and BMR-base excision repair). However, many other pathways also contribute to developing resistance to TMZ which includes hyperactivation of DNA repair pathways, aberrant signaling pathways, epigenetic modifications, autophagy, extracellular vesicle production, and microRNAs (Singh et al., 2021; Tomar et al., 2021). Poly (ADP-ribose) polymerase (PARP) inhibitors have been recently investigated as sensitizing drugs to enhance TMZ potency. PARP is a class of enzyme that participates in the BER pathway and is also involved in the MGMT pathway by physically interacting with and ultimately PARylates MGMT as a response to TMZ chemotherapy to eliminate adducts of O^6^-methylguanine (O^6^-MetG) from the damaged segment of DNA. Second, PARP works as a sensor, triggering the BER response pathways. PARP inhibitor drugs block binding of PARP-MGMT or PARylation of MGMT, reducing MGMT function and preventing O^6^-MetG repair. As a result, the MGMT function is reduced, resulting in TMZ sensitization and providing a rationale to sensitize (Wu et al., 2021). PARP inhibitor drugs have been investigated in GBM patients in a number of clinical trials. However, multiple factors make the clinical development of these inhibitors challenging. This review elucidates PARP and the relevance of PARP inhibitor drugs in the treatment of TMZ resistance in GBM. Furthermore, the challenges that may arise during the clinical trials of PARP inhibitors as well as the strategies to overcome these hurdles are highlighted.

## 2 Role of Poly (ADP-Ribose) Polymerase Inhibitors in Temozolomide Resistance

### 2.1 Temozolomide and Its Resistance in Glioblastoma Multiform

TMZ is a type of prodrug which is an imidazotetrazine analog of an anticancer alkylating agent, dacarbazine. This is commonly used as a chemotherapeutic agent under the brand name Temodar (Moody and Wheelhouse, 2014). TMZ is lipophilic in nature, and it can penetrate the BBB and be taken orally (Lee, 2016). It is a DNA alkylating agent that causes arrest at the G2/M phase of the cell cycle and subsequently induces apoptosis (Alonso et al., 2007). At physiological pH, TMZ drug is being transformed into 5-(3-methyltriazen-1-yl) imidazole-4-carboxamide (MTIC), a form of an active metabolite which is again degraded in 5-aminoimidazole-4-carboxamide (AIC) or methylhydrazine. The cytotoxic response of TMZ relies mainly on its DNA methylation efficacy, which takes place at the N7 or O6 guanine position or at the O3 position of adenine within the genomic DNA. Methylation at O6 guanine causes the addition of thymine rather than cytosine in front of methylguanine during the upcoming DNA replication process which further triggers the death of tumor cells (Lee, 2016). TMZ has been shown to be effective against human malignancies such as astrocytomas and melanomas (Middleton et al., 2000; Quirt et al., 2007; Yung et al., 1999; Hart et al., 2013). In 1999, TMZ was the first approved medicament used for recently diagnosed glioblastoma treatment as well as in refractory anaplastic astrocytoma in young patients by the United States Food and Drug Administration (USFDA). Patients who are newly diagnosed with adult GBM, when received concurrent Temodar and radiation, had shown a higher overall survival count than those who received radiation only (12.1/14.6 month’s average survival) (Cohen et al., 2005). Therefore, the currently available treatment for GBM is removal via surgery and subsequently by RT with adjuvant TMZ. The first-line chemotherapeutic agent TMZ is used for the treatment of GBM (Gupta et al., 2019; Singh et al., 2021). Unfortunately, due to the resistance, only around 50% of patients respond to TMZ (Lee, 2016). The principal pathway involved in the development of resistance of TMZ is through activation of various DNA repair pathways (MGMT, MMR, and BMR). However, many other pathways also play a vital role in TMZ resistance which includes hyperactivation of DNA repair pathways, aberrant signaling pathways, epigenetic modifications, autophagy, extracellular vesicle production, and microRNAs (Singh et al., 2021; Tomar et al., 2021). Recently, poly (ADP-ribose) polymerase (PARP) inhibitor drugs are being investigated as sensitizing drugs to enhance TMZ potency (Wu et al., 2021).

### 2.2 Poly (ADP-Ribose) Polymerases

**FIGURE 1:**
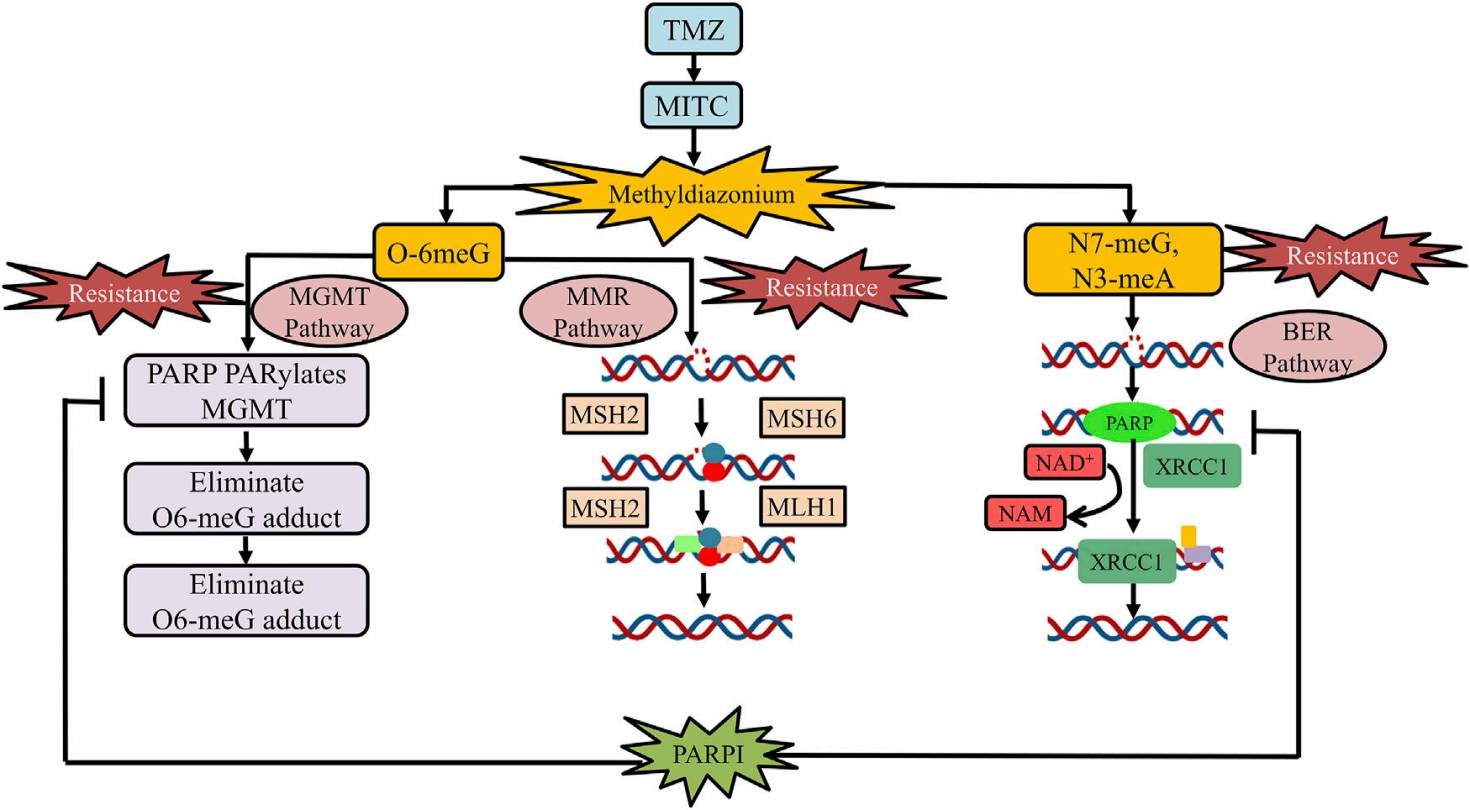
Role of PARP inhibitor drugs in TMZ resistance. At physiological pH, TMZ will be converted into MTIC and this MTIC is further hydrolyzed to methylhydrazine. The cytotoxicity of TMZ drug depends on the methylating/alkylating ability of methylhydrazine at the N7 or O6 positions of guanines or the O3 position of adenines in genomic DNA. Methylation at O6 position of guanine causes the addition of thymine rather than cytosine nucleotide opposite to methylguanine during the subsequent process of DNA replication, which triggers the death of tumor cells. The principal pathway involved in TMZ resistance development is the activation of DNA repair pathways (MGMT, MMR, and BMR). PARP is a class of enzyme that is being involved in the MGMT pathway by physically interacting with and PARylates MGMT as an effect of TMZ treatment to eliminate adducts of O^6^-MetG present in the damaged DNA strand. PARP inhibitors usually reduce the binding of PARP-MGMT along with PARylation as well as silencing of MGMT expression for O^6^-MetG repair. Hence, diminishing MGMT activity and rendering sensitization to TMZ. In the MMR pathway, the O6-meG:C pair gets mismatched after the first round of replication and results in O6-meG:T formation in the progeny DNA. The MMR system repairs the mismatch of O6-meG:T, by detaching the thymine-containing patch of newly generated strand, utilizing the number of complex proteins such as MSH2, MSH6, MLH1, and PMS2, respectively. Furthermore, PARP is a part of the BER complex that comprises XRCC1 protein, DNA ligase, and the DNA polymerase beta and is involved in BER in response to SSBs. PARP inhibitors reduced PARP binding in the BER complex, thus, reducing BER function to repair SSBs. Abbreviation: PARPI, PARP inhibitor; TMZ, temozolomide; MGMT, O6-methylguanine-DNA methyltransferase; MTIC, 5-(3-methyltriazen-1-yl)imidazole-4-carboxamide; MMR, mismatch repair; O6-MetG, O6-methylguanine; BMR, base excision repair; N7-meG, N7 methylguanine; N3meA, N3 methyl alanine; O6-meG:C, O6-methyl guanine:cytosine; O6-meG:T, O6-methylguanine:thymine; SSBs, single-stranded DNA breaks.

PARPs are a new class of enzymes that use β-NAD+ as a substrate to catalyze transmission of ADP-ribose on target proteins (i.e., poly ADP-ribosylation) (Amé et al., 2004; d'AMOURS et al., 1999; Lal and Snape, 2021). The process of attaching ADP-ribose to target proteins through PARP is known as PARsylation (Lal and Snape, 2021). PARPs are implied in a variety of processes at the molecular level, such as transcription, recombination, replication, DNA repair, and chromatin structure modulation (Morales et al., 2014). The PARP family comprises 18 members, encoded by a distinct gene and has a fixed catalytic domain. Some members of the PARP family such as PARP1 or PARP2 are widely recognized for their roles in DNA repair mechanism (Amé et al., 2004). PARP is a member of the BER complex, which includes XRCC1 protein, DNA ligase, and the DNA polymerase beta, and is involved in the BER-mediated pathway in response to single-stranded DNA breaks (SSBs) (Caldecott et al., 1996). In cell-free conditions, it has been found that the PARP enzyme in an unmodified form, attaches strongly to DNA strand breaks and, subsequently, auto-poly ADP-ribosylation is released, allowing repair enzyme exposure to access the damaged DNA (d'AMOURS et al., 1999; Satoh and Lindahl, 1992). Both PARP1 and PARP2 act as well as share similar partners in the SSR repair mechanism and BER processes (Amé et al., 2004; Schreiber et al., 2002). PARP1 has been found to play a functional response in nucleotide excision repair (NER), as NER functions are diminished when the PARP1 enzyme is being inhibited (Flohr et al., 2003). PARP is also involved in the MGMT pathway by physically interacting with it and ultimately PARylates MGMT as an effect of TMZ treatment to eliminate the O^6^-MetG complex in double-stranded damaged DNA, independent of the BER pathway (Wu et al., 2021). BER, NER, and MGMT are important pathways for repairing damaged DNA induced by alkylating and chemotherapeutic drugs. (15,103) Figure 1 elaborates on the role of PARP inhibiting drugs in TMZ resistance.

### 2.3 Poly (ADP-Ribose) Polymerase Inhibitors in Other Cancers

The PARP inhibitors are promising therapeutic agents involved in the treatment of different types of cancers. They exclusively produce synthetic toxicity in cancerous cells through homologous recombination deficiencies (HRDs), and one of the most prominent mechanisms is via mutations in the BRCA1/2 genes present in cancer cells. Current clinical studies suggest that PARP inhibitors can play a beneficial role in cancer therapy irrespective of BRCA1/2 or HRD status (Kim et al., 2021). Initially classified as an HR deficient cancer treatment, olaparib has been approved by FDA for the treatment of serous ovarian cancer as well as breast cancer having a mutation in BRCA1 or BRCA2 germline (Min and Im, 2020). Currently, the FDA has approved four PARP inhibiting drugs (olaparib, rucaparib, talazoparib, and niraparib) to ensure the treatment of breast carcinoma (having detrimental BRCA mutation) along with advanced ovarian cancer (Kim et al., 2021; Min and Im, 2020). The FDA, in May 2020, has also approved rucaparib and olaparib for young patients who are diagnosed with metastatic castration-resistant prostate cancer (mCRPC), having deleterious or suspected deleterious germline or mutation in somatic homologous recombination repair (HRR) gene (Maughan and Antonarakis, 2021). Along with these studies, olaparib has also been approved as a maintenance drug, used in germline BRCA1/2 mutant advanced PDAC (pancreatic ductal adenocarcinoma) by the United States FDA as a phase III Pancreatic Cancer Olaparib Ongoing (POLO) trials have revealed that the administration of olaparib (as a maintenance treatment) magnified the progression-free survival (PFS) rate when compared to a placebo (Chi et al., 2021; Zhu et al., 2020). The role of multiple PARP inhibitors has also been evaluated in gastric cancer (GC) conditions as mutated homologous DNA recombination genes such as BRC1/2, PALB_2_, ATM, RAD_51_C, and ARID_1_A carry somatic HRD (Sahasrabudhe et al., 2017). Therefore, various PARP inhibitors either in the form of mono (olaparib, talazoparib, pamiparib, rucaparib, and niraparib) or combinational therapy (olaparib + paclitaxel, olaparib + ramucirumab, and ceralasertib + olaparib) have gone under multiple phases of clinical trials. Also, FDA has approved ramucirumab in the treatment of gastroesophageal union adenocarcinoma or GC (Wang et al., 2021). In addition to these findings, a contrary role of PARP inhibitors has been reported in AML (acute myeloid leukemia)/MDS (myelodysplastic syndrome) conditions as microsatellite instability was linked to the reduced expression of HR repair genes (Kontandreopoulou et al., 2021). A phase I clinical trial data have shown a tolerable response produced by talazoparib (a PARP inhibitor), with decitabine, a DNA methyltransferase inhibitor when used against relapsed/refractory AML (Baer et al., 2022). On the other hand, a review of randomized controlled trials has reported that the use of different PARP inhibiting agents such as olaparib, niraparib, rucaparib, veliparib, and talazoparib significantly enhances the risk of AML/MDS in comparison to placebo (Kontandreopoulou et al., 2021). Also, mutation of the IDH1/2 gene in primary AML cells manifested HR abnormalities as well as a reduction in the expression of ATM, making AML cells PARP inhibitors susceptible. IDH1/2 inhibitors guard the cells from PARP inhibitors because DNA damage is reduced due to restoration of ATM expression. As a result, in IDH1/2 mutant AML, the use of PARP inhibitors with IDH1/2 inhibitors should be prevented (Molenaar et al., 2018). Notably, tumors without mutated HR-related genes (such as small-cell lung malignancies) have shown some susceptibility toward PARP inhibitors, potentially because of enhanced replication stress caused by RB1 mutations (Dias et al., 2021).

### 2.4 Poly (ADP-Ribose) Polymerase Inhibitors in Glioblastoma Multiform

In addition to other cancers, PARP inhibitors have demonstrated a significant response in various preclinical and clinical trials of glioma. Multiple studies investigated the efficacy of PARP inhibitors as they potentiate the anticancer activity of chemotherapeutic drugs and radiation when used simultaneously (Lal and Snape, 2021). Examples of actively acting PARP inhibitors against GBM, with their preclinical significance, are summarized in Table 1 while Table 2 comprises the PARP inhibitors which are effective against GBM, as well as their clinical status.

**TABLE 1:** Examples of actively acting PARP inhibitors against GBM, with their preclinical significance.

Drug (compound)	Clinical significance (comments)	Reference
Olaparib	Improved radiosensitivity in glioma cell lines (T98G, UVW, and U-373G). Improved radiosensitivity of pediatric high-grade glioma, ependymomas, and medulloblastoma cell lines. On the other hand, it is found to be a substrate for ABC transporters and has poor BBB penetration	Dungey et al. (2008) van Vuurden et al. (2011) Halford et al. (2017) and Vaidyanathan et al. (2016)
Rucaparib	Shown to be effective in sensitizing irinotecan and TMZ resistant tissues in human GBM xenografts without potentiating the myelotoxic effects. Also shown efficacy in sensitizing neuroblastoma or noradrenaline transporter expressing glioma cells to radiation exposure. Reported complete regression of tumor for more than 60 days when given in combination with TMZ. It is, however, found to be a substrate for ABC transporters and has poor BBB penetration in GBM murine xenografts	Miknyoczki et al. (2007) Nile et al. (2016) Thomas et al. (2007) and Calabrese et al. (2004) Parrish et al. (2015)
Niraparib	When given along with RT, it gradually improved mice survival rate in brain tumor models of pediatric high-grade astrocytoma and diffuse intrinsic pontine glioma. In phase I clinical trials, it was reported to be safe in advanced solid tumor patients	Chornenkyy et al. (2015) Sandhu et al. (2013)
Talazoparib	Reported sensitivity toward EFGR-amplified glioma sphere-forming cells. In tumors with DNA repair deficiencies, it has been observed to have synergistic action along platinum-based drugs and TMZ. Due to efflux by ABC transporters, brain permeability in GBM xenografts models is limited	Wu et al. (2020) Sachdev et al. (2019) and Shen et al. (2013) Kizilbash et al. (2017)
Veliparib	Reported highly effective in combination with TMZ in PTEN-deficient GBM mouse models. Given that the PTEN mutation is found in 36% of GBM patients, this is truly incredible. Also found effective in the treatment of patient-derived xenograft models and MGMT-unmethylated GBM cell lines when given along with RT. Due to the risk of reflux, there is moderate brain penetration with a brain-to-plasma ratio of 50%	Lin et al. (2014) and Wagner (2015) Jue et al. (2017) Gupta et al. (2016)
Pamiparib	Shown excellent brain permeability in animal studies and improved survival time when combined with TMZ.	Tang et al. (2015)
CEP-9722	Administered as a prodrug that has better deliverability, oral absorption as well as solubility and converted to CEP-8983 within 5 min of administration. Shown to be effective in sensitizing TMZ chemoresistance RG2 rat in GBM tumor model. In addition, when CEP-9722 was given 1 hour after TMZ, it diminished tumor progression by approximately 60% compared to TMZ administration alone, which only diminished tumor progression by approximately 32%	Miknyoczki et al. (2007)
E7016	In a phase I study, when co-administered with TMZ, it inhibited PARP activity and increased DNA damage in patients with advanced solid tumor. Reported increased tumor tissue sensitivity to radiation *in vitro* and *in vivo* by inhibiting DNA repair mechanisms in mice bearing U2521 xenografts	LoRusso et al. (2011) Russo et al. (2009)
A966492	When administered with 1 mM topotecan as well as irradiation, spheroids of U87 glioma cells showed radiation sensitivity of 1 mM. Reported lower selectivity for PARP-1 and PARP-2 than that of veliparib but greater than that of niraparib. In animal models, there is a high level of brain permeation	Koosha et al. (2017) Thorsell and Schüler (2017) Penning et al. (2010)
GPI 15427	Reported to enhance the survival rate when treated with TMZ in SJGBM2 glioma mice and also reduced tumor infiltration into other healthy tissues of mice. Shown to increase apoptosis and reduce tumor volume when given prior to radiation in tumor models	Tentori et al. (2003) Khan et al. (2010)
BTH-8	Reported inhibitory activity against the tumor growth of U87 GBM cell lines with an IC50 of 7.78 ± 1.68 Mm and also reported significant anti-tumor effect against numerous tumor cell lines and also *in vivo* tumor models by inducing apoptosis, arresting the cell cycle, and causing DNA DSBs	Guo et al. (2020)

**TABLE 2:** PARP inhibitor drugs which are under investigation in clinical trials and are active against GBM.

PARP inhibitor	Aim of the study	Objective of the study	Clinical trial number
Olaparib	A study of pembrolizumab, olaparib, and TMZ in glioma individuals at phase II. A phase II clinical trial of olaparib in IDH-mutant subjects having recurrent high-grade type gliomas A phase II investigation of olaparib with durvalumab (MEDI 4736) in IDH-mutant patients with solid tumors. There are three cohorts in this study: glioma with an IDH mutation, cholangiocarcinoma with an IDH mutation, and other solid tumors with an IDH mutation. A phase 2 trial of olaparib in advanced IDH1/2 mutant glioma, cholangiocarcinoma, or solid tumors. In a randomized phase 2 trial, olaparib and cediranib were evaluated by comparing to bevacizumab in patients with recurrent glioblastoma who had not previously received vascular endothelial growth factor (VEGF) therapy. Evaluation in phase I/IIa of olaparib and TMZ along with RT (in combination) to subjects with unresectable high-grade gliomas	To determine the safety and effectiveness of pembrolizumab, olaparib, as well as TMZ in combination; to see how well these drugs work when given together in people with glioma who either did not respond to earlier treatment or came back after treatment. The goal of this study was to see how effective olaparib is in IDH-mutant subjects with recurrent high-grade type gliomas based on their 6-month growth-free survival rate. To evaluate the effectiveness of combination therapy by focusing at the average response rate and disease prevention rate. It is thought that combining durvalumab and olaparib will be more beneficial to IDH-mutant patients with solid tumors than either drug alone. This study examines the effectiveness of olaparib in treating cholangiocarcinoma, glioma, and solid tumors which have a mutation at the IDH1 or IDH2 gene; unable to treat or control with current treatments; have migrated to other parts of the body. This randomized phase II trial compares the efficacy of olaparib and cediranib maleate to bevacizumab in treatment with recurrent GBM. Treatment through monoclonal antibodies (e.g., bevacizumab) may aid the immune system in attacking cancer while also interferes in tumor cells’ potential to proliferate and spread. Since normal brain cells do not divide, combining RT with PARP inhibitors (inhibitors of replication-specific DNA repair pathways) can improve the condition by increasing the cytotoxic effects of alkylating agents like TMZ	NCT05188508 NCT03561870 NCT03991832 NCT03212274 NCT02974621 NCT03212742
Niraparib	A phase 2 trial performed to evaluate the effectiveness as well as safety of niraparib with tumor treating fields (TTFields) in subjects with recurrent GBM. Measures safety and effectiveness of niraparib with addition to RT in treating recurrent GBM. A phase 0 “trigger” study of niraparib in newly diagnosed GBM and recurrent IDH1, IDH2, and ATRX mutant glioma	Examines the safety and effectiveness of niraparib with TTFields in individuals having recurrent GBM. Examines the safety as well as the effectiveness of niraparib in combination with RT within individuals having recurrent GBM. Measures effectiveness of niraparib in patients who have diagnosed with GBM recently and patients with IDH mutation and ATRX loss is being evaluated in this phase 0 studies with an expansion phase	NCT04221503 NCT04715620 NCT05076513
Talazoparib	Talazoparib in conjugation with carboplatin in subjects having recurrent high-grade glioma along with deficiency in the pathway of DNA repair	The purpose of this study is to see if talazoparib works in a glioma group with enriched biomarkers, as well as to see how a combinational treatment strategy affects patients, having recurrent type high-grade glioma that has a DNA repair pathway deficiency	NCT04740190
Veliparib	A phase 2 trial of veliparib (ABT-888) and local exposure to radiation preceded by veliparib with TMZ as a maintenance therapy, in recently detected high-grade glioma patients without mutations in BRAFV600 and H3 K27M. A randomized trial of phase II/III using placebo or veliparib with TMZ (combination) in GBM patients who are newly diagnosed with hypermethylation of MGMT promoter	Examines the combination effect of veliparib and TMZ as well as RT in recently detected malignant glioma without BRAFV600 or H3K27M mutations. Compares the efficacy of veliparib and TMZ (combination) versus TMZ alone in the treatment of newly diagnosed GBM.	NCT03581292 NCT02152982
Pamiparib	Phase 0/2 assessment of pamiparib in newly diagnosed and recurrent GBM	Investigated the response of pamiparib in newly diagnosed (unmethylated MGMT promoter) and recurrent GBM	NCT04614909

#### 2.4.1 Preclinical Studies of Drugs Targeting PARP-1 in Glioma

In 2008, a preclinical study by F. A. Dungey et al. showed that olaparib improves the radiosensitivity in glioma cell lines (T98G, UVW, and U-373G). The findings also reveal that olaparib improves radiosensitivity in a replication-dependent way which was increased by fractionation (Dungey et al., 2008). In addition, van Vuurden et al. reported a preclinical study in 2011 that showed olaparib improved radiosensitivity in pediatric high-grade glioma, ependymomas, and medulloblastoma cell lines. Also, gene expression profiling of pediatric high-grade glioma, ependymoma, and medulloblastoma showed that high expression of PARP1 has been linked to a poor prognosis (van Vuurden et al., 2011). Furthermore, a preclinical study by Miknyoczki et al. in 2007 explored another PAPP-1 inhibitor rucaparib which was reported effective in sensitizing irinotecan and TMZ resistant tissues in human GBM xenografts without potentiating the myelotoxic effects (Miknyoczki et al., 2007). A combination study of PARP inhibitors was performed by Nile et al. in 2016. This combined preclinical study elucidated the efficacy of olaparib and rucaparib in sensitizing neuroblastoma or noradrenaline transporter expressing glioma cells to radiation exposure. Rucaparib and olaparib were found to be equally potent inhibitors of PARP-1 functioning. PARP-1 inhibitors were used in combination with X-rays, and DNA damage was gradually increased 10-fold 2 h following irradiation (Nile et al., 2016). Another study of rucaparib by Calabrese et al. reported complete regression of tumor for more than 60 days when given in combination with TMZ. Rucaparib was found to be retained in xenograft tumors where PARP-1 activity was inhibited up to 70% for at least 4 h (Calabrese et al., 2004). Another PARP-1 inhibitor, niraparib, has been shown to be effective in both *in vitro* and *in vivo* models. Reduced rate of DNA damage repair, formation of the colony, and relative cell count were observed in an *in vitro* model before RT. pHGA cells were pretreated with a niraparib sublethal dose of 1 mol/L, and when given prior RT, *in vivo* niraparib (50 mg/kg) reduced PARP1 activity and increased mice survival rate in orthotopic xenograft model of pHGA (Chornenkyy et al., 2015). Another PARP-1 inhibitor, talazoparib, increased DNA damage and PARP–DNA trapping, enhancing cytotoxicity against EGFR-amplified glioma sphere-forming cells, Additionally, it reduced tumor growth significantly in EGFR-amplified subcutaneous models but not in nonamplified models (Wu et al., 2020). Shen et al. reported that talazoparib specifically targeted tumor cells with BRCA2, PTEN, or BRCA1 gene mutations with 20- to 200-fold better efficacy than current PARP1/2 inhibitors. Talazoparib showed a significant anticancer effect *in vivo* against xenografted tumors with DNA repair deficiencies caused by BRCA mutations and PTEN loss and was found to be sensitive to well-tolerated doses of talazoparib. Additionally, when talazoparib was administered in combination with TMZ, SN38, or platinum-based drugs, it had additive or synergistic antitumor effects (Shen et al., 2013). PARP-1 inhibitor veliparib was found to be highly effective in combination with TMZ in PTEN-deficient GBM mouse models. Given that the PTEN mutation is found in 36% of GBM patients, this is truly incredible (Lin et al., 2014). In addition, Jue et al. found that combining veliparib and RT inhibits the formation of the colony and increases apoptosis in most patient-derived cell lines. Furthermore, in a PDX model of MGMT unmethylated GBM, treatment with veliparib in a dose of 12.5 mg/kg in combination with complete brain RT (4 Gy) caused apoptosis and reduced cell proliferation. When compared to RT alone or veliparib alone, PDX treated with the combined treatment had significantly higher survival rates (Jue et al., 2017). In a study by Tang et al., pamiparib showed excellent brain permeability in animal studies and improved survival time when combined with TMZ. The *in vitro* combined effect of pamiparib and TMZ in 7 small-cell lung cancer (SCLC) and 8 GBM cell lines were investigated in this study. Pamiparib showed a significantly greater synergistic effect with TMZ in the majority of those cell lines. In addition, pamiparib showed considerable brain infiltration in C57 mice. To study the combination of pamiparib and TMZ on SCLC in the brain, mice models with established H209 xenografts (intracranial) were used. PARylation in brain or tumor tissues were significantly inhibited 4 h after 3 mg/kg single unit oral dose pamiparib. In this intracranial model, the addition of pamiparib dramatically increased animal survival compared to TMZ alone (Tang et al., 2015).

#### 2.4.2 Clinical Studies of Drugs Targeting PARP-1 in Glioma

A phase II clinical study of pembrolizumab, olaparib, and TMZ is now underway to determine the safety and effectiveness of these drugs when given in combination in glioma patients who failed to respond to earlier treatment or relapse. (NCT05188508) Another phase II clinical trial of olaparib in IDH-mutant subjects having recurrent high-grade type gliomas is completed, and this study elucidated the treatment efficacy of olaparib in IDH-mutant subjects with recurrent high-grade type gliomas based on their 6-month growth-free survival rate. (NCT03561870) A phase II investigation of the combination of olaparib and durvalumab (MEDI 4736) in IDH-mutant patients with solid tumors is nearing completion at the University Health Network in Toronto. There are three cohorts in this study: glioma with an IDH mutation, cholangiocarcinoma with an IDH mutation, and other solid tumors with an IDH mutation. Researchers hypothesized that combining durvalumab and olaparib will be more beneficial to IDH-mutant patients with solid tumors than either drug alone. (NCT03991832) Another phase 2 trial of olaparib in advanced IDH1/2 mutant glioma, cholangiocarcinoma, or solid tumors is also under completion. This study examines the effectiveness of olaparib in treating cholangiocarcinoma, glioma, and solid tumors which have mutations in the IDH1 or IDH2 gene; unable to treat or control with current treatments; have migrated to other parts of the body. (NCT03212274) In a randomized phase 2 trial, olaparib and cediranib combination was evaluated by comparing it to bevacizumab in patients with recurrent GBM who had not previously received vascular endothelial growth factor (VEGF) therapy. This randomized trial compares the efficacy of olaparib and cediranib maleate to bevacizumab in treatment with recurrent GBM. Treatment through monoclonal antibodies (e.g., bevacizumab) may aid the immune system in attacking cancer while also interfering with tumor cells’ potential to proliferate and spread. (NCT02974621) Evaluation in phase I/IIa of olaparib and TMZ along with RT (in combination) to subjects with unresectable high-grade gliomas. Scientists hypothesized that since normal brain cells do not divide, combining RT with PARP inhibitors (inhibitors of replication-specific DNA repair pathways) can improve the condition by increasing the cytotoxic effects of alkylating agents like TMZ. (NCT03212742) Niraparib has been established as an effective drug to treat ovarian cancer, and multiple clinical studies have reported an increased progression-free survival rate post therapy with niraparib. A phase II randomized trial with an estimated enrolment of N = 30 patients aged > 22 years has been hypothesized to evaluate the efficacy and safety of niraparib as primary outcomes and estimation of overall survival and progression-free survival as secondary outcomes [NCT04715620]. A phase I multicentered study by Kurzrock et al. has evaluated the tolerability and efficacy of niraparib and TMZ in individuals (N = 19) with recurrent GBM and melanoma, thrombocytopenia, progression of neoplasm, and leukopenia as major reported adverse events. At 40 mg, niraparib has shown antitumor activity (Kurzrock et al., 2014). Phase II study was executed to study efficacy and toxicity of veliparib in patients N = 66 with diffuse intrinsic pontine glioma. Veliparib 25 mg/m^2^ was added to the therapy along with chemotherapy. Grade 3 nervous system disorder, maculopapular rash, and hemorrhage inside the tumor were found to be major toxic effects, and there was no significant survival benefit compared to the control group (Baxter et al., 2020). A phase I tolerability study by Baxter et al. explored veliparib as maintenance therapy in combination with TMZ in pontine glioma patients N = 18. Veliparib 62 mg/m^2^ was well tolerated in the study population (Baxter et al., 2020). Ptirowski et al. (2019) concluded pamiparib 60 mg in combination with TMZ and radiation therapy was very well tolerated in GBM patients, with nausea and thrombocytopenia as major reported adverse events related to treatment. Recently, in 2021, the University of Hong Kong has initiated a study (NCT04740190) to explore the treatment efficacy of talazoparib and carboplatin combination in severe grade glioma patients.

#### 2.4.3 Poly (ADP-Ribose) Polymerase Inhibitors and Mechanism in IDH1/2 Mutant Cancer Type

IDH1/2 gene mutation was first recognized in the AML as well as glioma and afterward in numerous other cancers. GBM, low-grade glioma (Yan et al., 2009), cholangiocarcinoma (Jiao et al., 2013), acute myeloid leukemia (AML) (Mardis et al., 2009), chondrosarcoma, and melanoma (Amary et al., 2011; Krauthammer et al., 2012) have all been linked to IDH1 mutations. According to Parsons et al. (2008), almost 12% of patients suffering from GBM have IDH1 gene mutation i.e., R132H type. Although other variants such as R132C, R132L, and R132S at codon 132 are also found (Parsons et al., 2008). The 2q33 position of the chromosome carries the IDH1 gene which encodes for the enzyme IDH1 (Balss et al., 2008). Various studies have reported spontaneous mutations in the NADP^+^-dependent IDH1 gene in glioma along with mutation in the IDH2 gene which is located on 15q26.1 chromosomal position with a frequent mutation at R172K (sometimes also at R172K, R172W, and R172M) of R172 (Horbinski, 2013; Yan et al., 2009). Also, as per WHO classification, GBM has been classified into two main types. One is IDH-wt (wild type) GBMs which comprise primary or *de novo* GBMs while another is IDH-mut (mutation) GBMs which are secondary or progressive GBMs (Kaminska et al., 2019). Therefore, within glioma patients, a mutation in the IDH1/2 gene promotes the growth as well as the progression of glioma. The conversion of isocitrate to α-ketoglutarate (αKG) is being catalyzed by these enzymes, i.e., IDH1/2 to produce nicotinamide adenine dinucleotide phosphate (NADPH), and due to neomorphic mutations, an oncometabolite, i.e., 2-hydroxyglutarate (2HG) is being generated. A study has previously reported that the accumulation of 2HG inhibits the DNA repair process mediated by the HR process which results in the condition known as “BRCAness,” which offers sensitivity to PARP inhibitors (Dang et al., 2009). On the basis of these results, a multicenter study has been conducted in which patients suffering from IDH1/2 mutated gliomas were administered with a monotherapy olaparib and has shown tolerable response (Fanucci et al., 2022). Also, as per preliminary findings, olaparib has been found to be safe as well as effective in IDH1/2-mutant mesenchymal sarcoma condition (Eder et al., 2021). Another *in vitro* research has evaluated the effect of PARP inhibitors in combination with ATR inhibiting molecules in IDH1/2-mutant cells and suggested that this combination therapy elevates the premature mitotic entry in the presence of IDH1/2 mutations (Sule et al., 2021). Various clinical studies are still ongoing in different phases in which PARP inhibitors are used in IDH mutated glioma patients. Niraparib (NCT05076513) is being evaluated in recurrent *IDH*-mutant glioma (cohort B) study, while many combinational drugs like pamiparib/TMZ (NCT03914742) and radiotherapy along with carboplatin/talazoparib (NCT04740190) are studied in high-grade glioma with *PTEN*-mutant, *IDH*-mutant, and BRCAness signatures (Sim et al., 2022). Therefore, PARP inhibitors are one of the promising as well as desperately needed domains to be explored more in the field of neuro-oncology research.

## 3 Challenges

**FIGURE 2:**
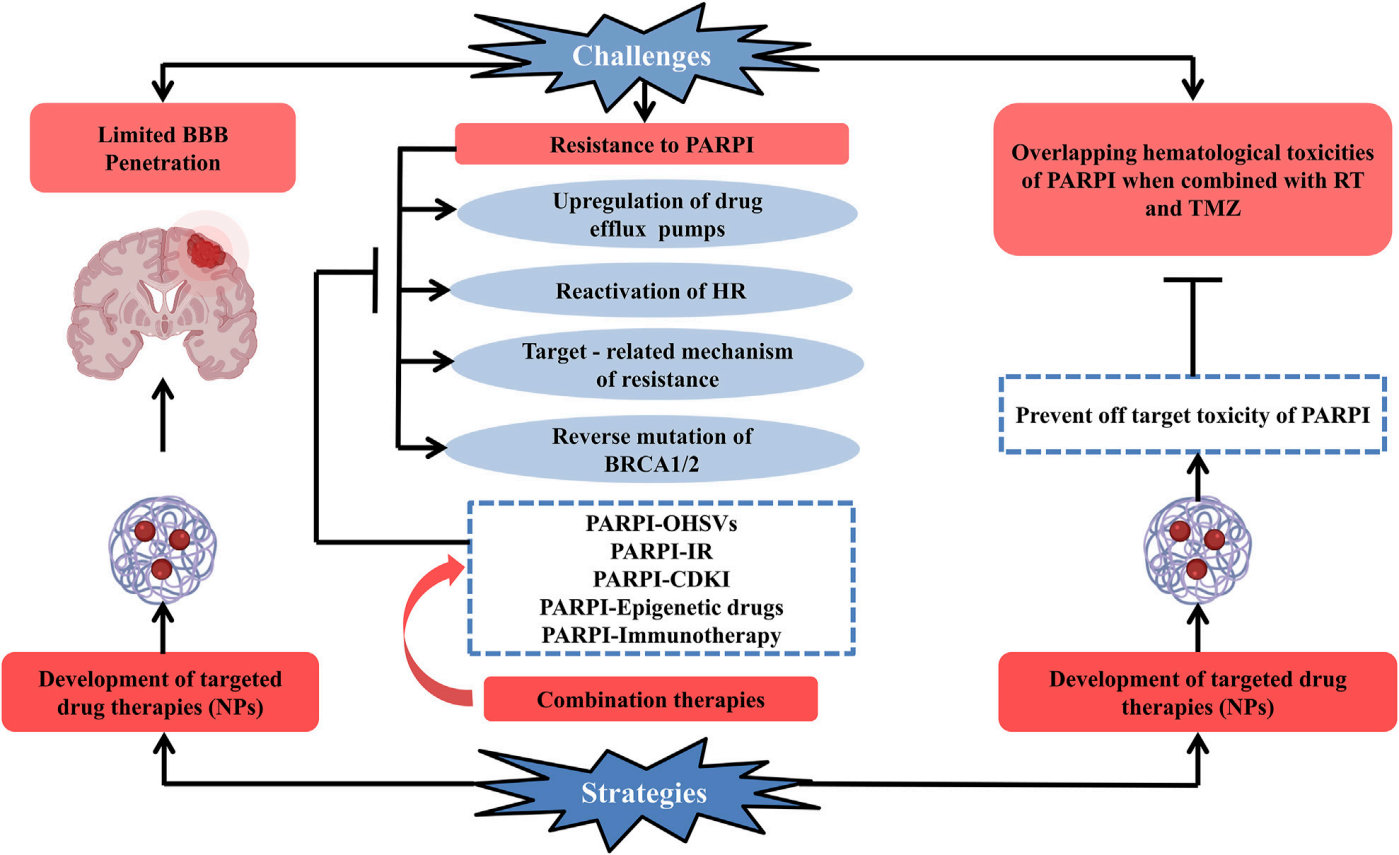
Schematic representation of current challenges along with the possible strategies which can be opted for the efficient clinical development of PARP enzyme inhibitors used for the treatment of glioblastoma. Development of PARP enzyme inhibitors for glioblastoma is clinically challenging due to multiple factors: limited blood–brain barrier penetration of PARP inhibitor drugs and development of resistance toward PARP inhibitors (upregulation of drug efflux pumps, reactivation of HR, targeted-related mechanism of resistance, and reverse mutation of BRCA1/2), overlapping hematologic toxicities of PARP inhibitor drugs when conjugated with glioblastoma’s standard treatment (radiation therapy + temozolomide). One of the promising strategies to deal with the challenges is the development of targeted drug therapies and combination therapy (PARPI-IR, PARPI-OHSVs, PARPI-CDKI, PARPI-epigenetic drugs, and PARPI-immunotherapy) to combat the resistance of PARP inhibitors to overcome these factors. Abbreviations: PARPI, PARP inhibitor; OHSVs, oncolytic herpes simplex viruses; IR, ionizing radiation; CDKI, cyclin-dependent kinase; NPs, nanoparticles.

Clinical development of PARP inhibitors against glioblastoma is quite difficult because of multiple factors (Figure 2). Some of them are enlisted as:

### 3.1 Limited Blood–Brain Barrier Penetration of Poly (ADP-Ribose) Polymerase Inhibitors

The targeted delivery of therapeutically active compounds into the CNS is a significant challenge in the treatment of most neurological diseases. The BBB is one of the most complex and well-protected neurovascular unit composed of tight junctions between endothelial cells and brain capillary, which restrict paracellular diffusion and protects the brain from harmful chemicals and toxins by separating it from peripheral circulation (Agarwal et al., 2012; Gupta et al., 2019). Thus, it limits the exclusion of many anticancer drugs including PARP inhibitors and undermines their effectiveness (Agarwal et al., 2012; Saran et al., 2018). In GBM, glioma cells can infiltrate normal brain tissue some centimeters apart from the tumor, evading surgical resection and so being secured by a relatively intact BBB (Berens and Giese, 1999; Pitz et al., 2011). Even though the BBB may be damaged at or around the tumor core, it stays intact in locations distant from the core, limiting anticancer drug delivery in these areas (Levin et al., 1980). This implies that in several GBM patients, restricted drug delivery to invasive tumor cells could play a crucial role in therapeutic resistance. This type of failure in drug delivery needs more exploration in preclinical models. Since some preclinical models are not developed in the brain such as the flank model, and some involve mice with well-circumscribed brain tumors obtained from implanted cell lines that demonstrate a leaky BBB without any significant invasion (Blakeley et al., 2009). Preclinical data involving GBM animal models imply that the efficiency of PARP inhibitors in GBM could be restricted because of limited transport over the BBB membrane and heterogeneous glioma response (Gupta et al., 2014; Kizilbash et al., 2017; Parrish et al., 2015; Gupta et al., 2016). Significant attempts had been made to acknowledge the brain pharmacokinetics of PARP inhibitors, and many PARP inhibitors, particularly the trapping agents like rucaparib and talazoparib, exhibit efflux liabilities around the BBB, and thus a complete absence of sensitizing response is observed in orthotopic models of tumor in spite of great activity in heterotopic cancer models (Hopkins et al., 2015; Parrish et al., 2015). These results support the hypothesis that targeted delivery of drugs within the healthy brain or orthotopically implanted tumor can simulate their potency in the GBM. Talazoparib, a PARP inhibitor, enhances the potency of temozolomide in a variety of tumor types. However, when the pharmacokinetic parameters of talazoparib were compared to another PARP in normal rodents, a low ratio of brain-plasma concentration of talazoparib (approximately 0.02) was observed, indicating that it lacks effectiveness in the orthotopic type of glioma models (Parrish et al., 2015; Kizilbash et al., 2017). Alternative PARP enzyme trapping compound bearing efflux liability as well as limited distribution around BBB is olaparib (Halford et al., 2017; Vaidyanathan et al., 2016). Despite the reality that olaparib was found to penetrate the center of GBM tumors in the phase I clinical trial in individuals with repetitive GBM (Halford et al., 2017), these findings must be considered with caution since GBM cells infiltrate tissues far away from the margins. Rucaparib in combination with TMZ was found to be extremely effective in a study performed in short explant cultures (*in vitro*) obtained from GBM12, and the same combination (when dosed for 5 days in a consecutive manner every 28 days for three cycles) significantly prolonged the tumor regrowth duration by at least 40% in heterotopic xenografts. However, a PK/PD investigation following a unit dose revealed that rucaparib deposition within the brain is related to elevate over left PARP enzymatic activity (Parrish et al., 2015). Conversely, veliparib seems to have a significantly greater ratio of brain-plasma concentration (around 0.47) rather than rucaparib and talazoparib in spite of its efflux liability toward MDR1 as well as BCRP (Gupta et al., 2016; Li et al., 2011). In addition, orthotopic GBM models are sensitized better from veliparib rather than rucaparib and talazoparib comparatively, although being much less powerful with respect to PARP trapping (Gupta et al., 2016). This premise sheds light on the importance of factors like penetrability across the BBB and efflux liability in orthotopic models of glioma which has a role to improve efficacy. These findings highlight the significance of neuronal pharmacokinetic parameters in preclinical models for the successful clinical development of innovative GBM therapies.

### 3.2 Resistance to Poly (ADP-Ribose) Polymerase Inhibitors

Although PARP inhibitor drugs impart a positive response initially, many subjects acquire resistance to them, resulting in disease recurrence. The emergence of resistance to PARP inhibitor drugs can occur via five basic mechanisms: overexpression of drug efflux pumps, target-related mechanism for resistance, restoration of BRCA1 or BRCA2 gene activity, BRCA1-independent restoration of HR, and restoration of fork stability.

#### 3.2.1 Upregulation of Drug Efflux Pumps

Overexpression of ABCB1 type transporters (also known as P-glycoprotein), which are mainly responsible for the efflux of drugs, has been reported among the earliest reasons for the generation of resistance to PARP inhibitor drugs. ABCB1 is a type of ATP-binding cassette (ABC) transporter, which has been shown to confer resistance to a variety of chemotherapeutic and many other drugs by limiting their accumulation intracellularly. The development of resistance against PARP inhibitor is being mediated by ABCB1, and it was first reported in BRCA1/2-deficient breast cancer mice models (Rottenberg et al., 2008). Patients who have received chemotherapy before starting PARP inhibitor treatment are more susceptible to this resistance mechanism, as ABCB1 transporters have been shown to be upregulated in tumors that are formerly exposed to chemotherapies because of chromosomal translocations that occur during treatment through paclitaxel anticancer agent (Marzolini et al., 2004). Interestingly, resistance may be overcome by ABCB1 inhibitors (verapamil and elacridar), which can be used against drug resistance mediated by ABCB1, in ovarian cancer treated by olaparib and paclitaxel (Vaidyanathan et al., 2016). Clinical trials with ABCB1 inhibitors have shown unsatisfactory results, and recent research indicated toward ABCB1 is crucial for effective immunological response (Chen et al., 2020). Further investigation is required to clarify the significance of ABCB1 gene screening in developing therapy regimens.

#### 3.2.2 Target-Mediated Mechanism of Resistance

Each available PARP inhibitor drug competes with coenzyme NAD+ to target the PARP enzymes at its catalytic domain site. Resistance could thus develop from PARP1 mutations that decide whether it decreases the efficacy of the PARP inhibitor compound or conserve primary in-built activities of the PARP enzymes when bound to the PARP inhibitor. *In vitro* results indicate that the development of resistance to PARP inhibitors, associated with point mutations is present not only in the enzyme’s catalytic region but also present in domains through which PARP1 is being trapped on the DNA strand (Pettitt et al., 2018; Pettitt et al., 2013). Confirming these findings, in a PARP inhibitor-resistant ovarian tumor, a mutated PARP1 was found that does not alter the mobilization of the PARP1 enzyme toward the damaged locus of DNA but prevents the trapping of PARP1 (Pettitt et al., 2018). However, mutations in PARP1 exclusively develop resistance in cells which are HR-proficient and with hypomorphic BRCA1 mutation in cells and synthetic lethal effect are produced which causes loss of both PARP1 and BRCA1 in response to BRCA1 activity at the residual level. Another enzyme poly (ADP-ribose) glycohydrolase (PARG) is an important factor that was found to play a pivotal role in the generation of resistance toward PARP inhibitors. This enzyme separates the PAR chain from the targeted protein. For example, in genetically modified models of BRCA1/2-deficient mice for breast carcinoma, depletion of PARG leads to the development of resistance to PARP inhibitor drugs. Significantly, in these models, PARG lacking cells treated to inhibit PARP are predicted to maintain enough PARylation at desired targeted proteins so that they can initiate the signaling cascade for DNA damage as well as minimize PARP1 protein trapping onto DNA strand due to remaining PARP activity. Despite the lack of clinical evidence, two small cohort studies reported that PARG negative areas accounting for 10% or more of the overall tumor mass which have been found in a significant proportion of tumors present in females suffering from triple-negative breast cancers (76.8%) or ovarian cancer (78.4%), for which PARP inhibitors can be used as a therapeutic approach for both (Gogola et al., 2018).

#### 3.2.3 Reverse Mutation of BRCA1/2

One of the clinically proven mechanisms for the development of resistance toward PARP inhibiting drugs is reverse mutation and modification at the epigenetic level, which causes the repetitive expression of a BRCA1/2 protein and leads to hypomorphic variations. After long-term treatment with PARP inhibitors or cisplatin, the mutation in reversion of protein-truncating BRCA1 or BRCA2 gene was first observed in an *in vitro* study performed in BRCA2 mutated gene in pancreatic as well as ovarian cancerous cell lines (Edwards et al., 2008; Sakai et al., 2008). Patient-derived xenograft (PDX) models of mutated BRCA1 gene or BRCA1 methylated gene in TNBC disclosed resistant after the exposure to PARP inhibiting drugs due to intragenic removal which reinstate the reading sequence of mutant BRCA1, and absence in BRCA1 gene promoter hypermethylation as well as *de-novo* gene fusions that results in upregulated expression of epigenetically silenced gene BRCA1 (Ter Brugge et al., 2016). Over many recent years, multiple investigations had identified that reversions of BRCA1/2 at the genetic level are a cause of PARP inhibitor resistance during ovarian (Edwards et al., 2008; Kondrashova et al., 2017; Weigelt et al., 2017; Barber et al., 2013; Norquist et al., 2011; Domchek, 2017; Lin et al., 2019), prostate (Quigley et al., 2017; Goodall et al., 2017), breast (Weigelt et al., 2017; Barber et al., 2013; Afghahi et al., 2017), and pancreatic cancers (Pishvaian et al., 2017). Interestingly, reversions linked with PARP inhibitor resistance are not only reported in BRCA1 and BRCA2 but have also been reported in some HR-related genes including PALB2 and RAD51C/1D (Kondrashova et al., 2017; Goodall et al., 2017). Due to the challenges of detecting reversion mutation and the limited usage of PARP inhibitors in clinical trials, data gathered from massive-scale research to assess the prevalence of BRCA1/2 gene reactivation in cancer patients having PARP inhibitor-resistant tumors are missing even now.

#### 3.2.4 Reactivation of Homologous Recombination

A mutation in the NHEJ (non-homologous end-joining) pathway results in the resurgence of homologous recombination (HR) in mutant cells of BRCA1/2. BRCA1 induces strand exchange through its interaction with BRCA2 and PALB2 in normal cells. Additionally, it antagonizes 53BP1 and impairs the NHEJ pathway (Isono et al., 2017). 53BP1 is an essential element of NHEJ machinery and imparts a crucial response in repairing DNA as well as checkpoint control (Bunting et al., 2010; Mirman and de Lange, 2020; Zimmermann and De Lange, 2014). It stimulates NHEJ through decreasing DNA end-resection process that is essential for HR. Furthermore, it interacts with a few genes such as RIF1, REV7 and SHLD1, SHLD2, and SHLD3 (shieldin complex) whose resulting complex known as 53BP1-RIF1-REV7-shieldin axis that prevents incision (Xu et al., 2015; Zimmermann et al., 2013; Noordermeer et al., 2018). The depletion of any of the components in this complex has been implicated in PARP inhibitor resistance in cells that are BRCA1 deficient but notably not in cells that lack the BRCA2 gene (Bouwman et al., 2010). It is hypothesized that the suppression of the shieldin axis, i.e., 53BP1-RIF1-REV7-axis, enables resection at the end and then HR to take place in an independent manner in BRCA1 while in a dependent manner in RNF168. RNF168 belongs to the E3 ubiquitin ligase category enzyme which can activate HR without any need for BRCA1 requirement by interacting directly with PALB2. Dynein light chain 1 (DYNLL1) is yet another molecule which has been associated to develop PARP inhibitor resistance, like 53BP1, acts by blocking the end resection of DNA in normal cells and promotes NHEJ. Although it has been reported that DYNLL1, which is a coding gene for protein, interacts with 53BP1 to limit terminal resection or that DYNLL1 gene which acts by blocking resection machinery elements like MRE11 (Lo et al., 2005; Becker et al., 2018; He et al., 2018), the actual relation between 53BP1 axis and DYNLL1 remains unknown. Similarly, inhibition of 53BP1 and inactivation of the DYNLL1 gene are linked to PARP inhibitor resistance (He et al., 2018). The deprivation of ERCC6L2, which is an additional NHEJ component, has been reported to reestablish DNA end-resection, leading to incomplete (half) HR repair and acquiring resistance toward PARP inhibiting compounds in cells lacking the BRCA1 gene (He et al., 2018; Francica et al., 2020). Furthermore, upregulation of factors that promotes HR and inhibits NHEJ, including TIRR134 (Drané et al., 2017), TRIP13 (Clairmont et al., 2020), and miRNA-622 (Choi et al., 2016), have been reported to protect HR plus diminish the sensitivity toward PARP inhibiting compounds in BRCA1 gene-deficient cells. Such results support the hypothesis which states that the induction of resistance to PARP inhibitor drugs develops from the failure of DNA end protection within functionally active BRCA1 diminished cells. Importantly, the foregoing data indicates that in the HR pathway mediated by RAD51, BRCA2 protein is critical for the pathway while BRCA1 protein is somewhat required for the last few steps in the same pathway.

#### 3.2.5 Reactivation of Fork Stability

Generation of resistance to PARP inhibitor drugs from reactivation of fork stability is frequent in both BRCA1- and BRCA2-deficient cells. As stated formerly, BRCA1/2 is needed not just for HR but, moreover, to maintain the stability as well as to provide protection to the replication forks during replicative strain. MRE11 or MUS81 are the two nucleases which are needed for the formation of halted replication forks. Uncontrolled excision of unprotected halted forks by MRE11 causes collapse of the fork and results in elevated instability at the genomic level in BRCA1/2-deficient cells (Schlacher et al., 2011; Schlacher et al., 2012; Ray Chaudhuri et al., 2016; Ying et al., 2012). In view of this fact, loss of protein, i.e. PTIP, a complex of MLL3/4 as well as nucleosome remodeling element CHD4 impairs MRE11 attachment in obstructed forks; resulting in protection of fork that leads to PARP inhibitor resistance in cells deficient in BRCA1/2 (Ray Chaudhuri et al., 2016; Guillemette et al., 2015). SMARCAL1, a chromatin remodeling complex depletion, reduces the sensitivity of tumor cells deficient in BRCA1/2 and causes PARP inhibitor resistance, however, this effect appears to be cell-type specific (Taglialatela et al., 2017; Kolinjivadi et al., 2017). Another protein associated with replication fork protection is RADX; reduction of this protein in cells deficient in BRCA2 restores protection of replication fork and reduces the toxic effects of PARP inhibitor compounds (Dungrawala et al., 2017). Also, preventing MUS81 gathering by inhibiting the methyltransferase activity of EZH2 induces protection of fork and partially PARP inhibitor resistance, especially in cells deficient in BRCA2 (Rondinelli et al., 2017). Unfortunately, there is conflicting evidence on the involvement of MUS81, with studies claiming that this nuclease either disrupts (Dungrawala et al., 2017; Rondinelli et al., 2017) or protects (Lai et al., 2017; Lemaçon et al., 2017) the unprotected forks; consequently, whether the cytotoxic response of PARP inhibiting drugs is affected by MUS81 within BRCA1 and BRCA2 diminished cells seems unknown (Lemaçon et al., 2017). Interestingly, loss of PTIP, RADX, or EZH2 does not improve the functioning of HR in cells having low BRCA1/2, implying that reactivation of protection of replication fork is a major aspect for induction of resistance toward PARP inhibitor compounds (Ray Chaudhuri et al., 2016; Dungrawala et al., 2017; Rondinelli et al., 2017). Another replication stress factor is Schlafen 11 (SLFN11) whose depletion lowers the cytotoxic response of PARP inhibiting compounds in BRCA1/2 proficient as well as BRCA2 diminished cells (Murai et al., 2016). Lastly, and perhaps most notably, PARP1 is required to facilitate MRE11 localization to a stalled replication fork. PARP1 deficiency causes synthetic toxicity in BRCA1/2 diminished cells; however, PARP1 repression prior to BRCA1or BRCA2 depletion re-establishes the integrity within the stalled forks and increases cell viability, widely through decreasing MRE11 localization at the replication fork (Ray Chaudhuri et al., 2016; Ding et al., 2016). Considering the multifaceted function of PARP1 at the replication fork, more research is needed to clarify how it may alter the outcomes of PARP inhibitor-based combination therapy.

### 3.3 Overlapping Hematologic Toxicities of PARP Inhibiting Drugs When Combined With Standard Treatment for Glioblastoma (RT and TMZ)

The acceptability of veliparib in conjugation with the standard treatment of newly diagnosed GBM (RT and TMZ) was investigated in a phase 1 trial. The study showed that a daily 10 mg dose (twice a day) of veliparib when administered orally, with concurrent RT/TMZ was intolerable in individuals suffering from GBM due to hematological toxicity (Kleinberg et al., 2013). A randomized study under phase 1/2 clinical assessment investigated a combination along with TMZ for treating persistent GBM with TMZ resistance. The myelosuppression with grade 3/4 was reported in approximately 20% of patients, with a usual progression-free survival (PFS) for 2 months (Robins et al., 2016). PARP inhibitors in addition to TMZ have also been studied in neuro-oncology and in the pediatric population. A phase I study of veliparib in conjugation with TMZ in the juvenile population suffering from recurrent brain tumors has been reported. Dose-dependent myelosuppression was observed in children (Su et al., 2014). Furthermore, in the phase I concentration escalation study, using olaparib in addition to TMZ within relapsed GBM patients was performed in the US. Pharmacokinetics study of orally bioavailable olaparib against tumor was studied after four doses using tumor resection. Olaparib penetrated tumors, with a mean concentration of 588 nM in the tumor core and 500 nM in the tumor tissues. When a daily dose of 150 mg of olaparib was administered for 1–3 days weekly in conjunction with 75 mg/m^2^ of TMZ, the response was well tolerated. Totally, 45% of the evaluable subjects were progression free following 6 months. However, 24 of the 35 evaluable patients reported adverse effects ≥3. The most frequent symptom was lymphopenia (51%), followed by neutropenia (26%), thrombocytopenia (17%), anemia (14%), and fatigue (Halford et al., 2017). This finding prompted us to conduct a clinical trial in GBM patients who are newly diagnosed and treated with olaparib in adjuvant to RT or RT + TMZ depending on MGMT status stratification (phase 1, PARADIGM-2). In this PARADIGM-2 study, within a single trial protocol, patients were allocated into two respective groups parallelly. Patients who are allocated within parallel 1 of the study (MGMT methylated) were administered olaparib in combination with radical RT (60 Gy in 30 fractions for 6 weeks) with concomitant TMZ chemotherapy (75 mg/m^2^ each day throughout RT), followed by 4 weeks of oral olaparib, subsequently followed by TMZ at its standard effective dose for six cycles and schedule commencing 4 weeks post radiotherapy. Patients assigned to parallel 2 of the study (MGMT unmethylated) received olaparib with radical RT in combination (60 Gy in 30 fractions for 6 weeks), and a further 4 weeks of oral olaparib. Hematological toxicities such as neutropenia grade 4 (lasting for ≥5 days), febrile neutropenia of grade ≥3 (absolute neutrophil count <1.0 × 10^9^/L with ≥38.5°C fever), and thrombocytopenia of grade 4 (lasts for ≥5 days) linked with heavy bleeding or requiring platelet transfusion reported during the treatment period of olaparib in both MGMT methylated and methylated patients (Fulton et al., 2018). A newer generation of PARP inhibitor BGB-290 (pamiparib) is now also being studied in clinical trials. A phase 1b/2 assessment was conducted in patients having first-line or recurrent/refractory GBM to evaluate the tolerability and safety as well as the effectiveness of BGB-290 with RT + TMZ (in conjugation). In a dose-escalation/phase 1b study, pamiparib was conjugated with RT (arm A) and RT plus TMZ (arm B) in recently detected unmethylated GBM patients while arm C of the test has pamiparib in conjugation with TMZ for methylated or unmethylated recurrent/refractory GBM patients. The dose expansion or phase 2 study enrolled up to four cohorts: recently detected unmethylated GBM patients in arm A as well as arm B, and cohorts of patients bearing recurrent/refractory GBM divided via MGMT status (unmethylated/methylated) within arm C. Patients of arms A or B have been treated till their RT is completed, while patients in arm C could undergo treatment if there are no safety concerns or tumor growth. BGB-290 at 60 mg BID in combination with RT/TMZ was well tolerated in recently diagnosed or recurrent/refractory GBM patients. However, one dose-limiting adverse event (grade 3 type febrile neutropenia) was observed in patients of arm B. Although, treatment-associated adverse effects (≥10%) were arm A experiences nausea (around 23 percent/2 percent); B reported a decrease in WBC count (approximately 11 percent/11 percent); C (none) (Piotrowski et al., 2019).

## 4 Strategies

### 4.1 Formulating Targeted Drug Therapies (Nanoparticles)

The development of nanoscale drug delivery vehicles including nanoparticles, liposomes, dendrimers, nanomicelles, polymeric micelles, liposomes, and dendrimers has completely transformed new drug delivery approaches. These nanotherapeutic techniques are widely implemented in clinics for the betterment of the efficacy of active small-molecule inhibitors due to their remarkable cancer-targeting efficacy and sustained release. PARP inhibitor drugs are small artificial molecules that have proven to be one of the most effective new approaches to treating carcinoma cells with alterations in repair genes of DNA. PARP inhibitor efficacy has been greatly enhanced due to the advancement of nanotherapeutic-based delivery of drugs. Nanoparticles could especially accumulate inside the tumor and cancer cell’s leaky vasculature, releasing the cytotoxic compound inside the microenvironment of the tumor cell. But on the other hand, nanoparticles will not be able to diffuse through the non-cancerous body tissues as well as organs, so their toxic response is minimal to non-existent. Targeting PARP inhibitors to treat cancer frequently results in toxicity; thus, a delivery carrier is required to encapsulate them (Sargazi et al., 2021). Several nanovehicles have now been used to transmit PARP inhibitor compounds in various cancerous cells (Mensah et al., 2019; Baldwin et al., 2018b; Di Zhang et al., 2019; Patel et al., 2017; Baldwin et al., 2018a). In a study, a nanoformulation of olaparib in conjugation with platinum was developed, and its activity was illustrated in the cell line of ovarian cancer. The ADME as well as bioavailability profiles were improved by the nanoformulation. They improved the therapeutic response by inhibiting cell proliferation (Baldwin et al., 2018b). In a different study, lipid-based nanoformulation was developed to deliver olaparib. The lipospheres are created through melt dispersion along with nanosuspension via wet processing or evaporation of the solvent. The ADME profiles or hematological adverse response of olaparib nanosuspension and lipospheres were compared. The nanoparticles had a high bioavailability and no toxicity in the tissues (Pathade et al., 2019). In another study, polymeric vehicles were used to develop drug carriers. Drug nanocrystals, pectin, and bioadhesive hydrogel were encapsulated with polylactic acid-polyethylene glycol and sprayed in the brain’s parenchyma to treat GBM. The nanoparticles gelled at the calcium concentration inside the brain. Both olaparib as well as etoposide were packed in the polymeric carrier synergistically, and for spray-drying purposes, pluronic F127 was used. Over the course of 120 h, the drug was released. The nanoparticles had aggregated in the brain according to fluorescent imaging results. A pharmacological platform for malignant brain cancers was introduced by the sprayable hydrogel (McCrorie et al., 2020). These simplistic but novel strategic approaches can boost the activity of PARP inhibitors.

### 4.2 Combating Poly (ADP-Ribose) Polymerase Inhibitors Resistance

One of the promising strategies to deal with the resistance of PARP inhibitors in tumor cell is combination therapy.

#### 4.2.1 Poly (ADP-Ribose) Polymerase Inhibitors With Oncolytic Herpes Simplex Viruses

Oncolytic herpes simplex viruses (oHSVs) are compounds synthesized through genetic modification to specifically destroy cancer cells because of their unique property of reproducing as well as propagating inside the malignant cells except for the normal ones. The FDA has approved oHSVs for the treatment of recurrent melanoma. They actively participate in DNA damage repair (DDR) manipulation (Kohlhapp and Kaufman, 2016). MG18L, a recently discovered activity of oHSV, has been shown to deteriorate RAD51 as well as sensitize GBM stem cells toward PARP inhibitors, by killing cells in an artificial lethal like manner both *in vitro* as well as *in vivo*. A combined effect of two, i.e., olaparib and MG181, significantly improves the condition in PARP inhibitor sensitive cells along with resistant GBM stem cells derived from tumor cells. Fused treatment (olaparib + MG181) not just combats PARP inhibitor resistance but it also broadens the application to malignancies along with HR-proficient. Especially, when compared to traditional treatments, oHSVs only destroy tumor cells by not affecting normal cells, and this implies that they could have minimal side effects (Ning et al., 2017).

#### 4.2.2 Poly (ADP-Ribose) Polymerase Inhibitors-Ionizing Radiation Combination

BRCA1 requires localization of the nucleus in order to participate in homologous recombination-mediated repair of DNA (Wang et al., 2010). Ionizing radiation can cause BRCA1 to be exported to the cytoplasm from the nucleus, increasing the sensitivity of PARP inhibitors in the wild-type BRCA1 and homologous recombination proficient tumor cells (Jiang et al., 2011; Yang et al., 2012). The combination therapy can be used only in patients with p53 wild-type because its synthetic lethality is p53-dependent (Sizemore et al., 2018). Furthermore, it was reported that tumors mutated due to the BRCA1 gene developed resistance as a result of BRCA1-independent homologous recombination restoration which can be sensitized to radiotherapy (Barazas et al., 2019). Based on preclinical findings, multiple clinical experiments were performed in order to optimize the potency of PARP inhibitors-ionizing radiation combination. A phase-1 concentration escalation study (open-labelled) in brain metastasis individuals to evaluate the combination of veliparib with whole-brain radiation therapy and found better results (Mehta et al., 2015). Furthermore, two phase-I trials concluded that the PARP inhibitors-ionizing radiation combination was well-tolerated and produced decent results (Reiss et al., 2015; Czito et al., 2017). More studies are currently underway on the basis of encouraging preliminary safety and efficacy outcomes.

#### 4.2.3 Poly (ADP-Ribose) Polymerase Inhibitors-Cyclin Dependent Kinase Inhibitors Combination

The end resection of DNA is dependent on the activity of cyclin-dependent kinases. Numerous studies have found that CDKs play prime roles in PARP inhibitor resistance (Tomimatsu et al., 2014; Ali et al., 2014; Joshi et al., 2014; Johnson et al., 2016; Ning et al., 2019; Militello et al., 2019). Dinaciclib, a CDK inhibitor, re-sensitized triple-negative breast cancer cells that had developed niraparib resistance. Furthermore, the combination of dinaciclib and niraparib was extremely effective in pancreatic, lung, colon, prostate, and ovarian cancer (Carey et al., 2018). Cyclin-dependent kinase-12 has recently gained more interest in PARP inhibitor resistance owing to its inactivating somatic alterations that have been found in various cancers. Several studies have shown that CDK12 mutation or deficiency causes cancer cells to be more sensitive to PARP inhibitors (Ali et al., 2014). In addition to this, cyclin-dependent kinase-12 inhibitors have reversed *de novo* and developed PARP inhibitor resistance in breast cancer cells mutated due to the BRCA1 gene (Johnson et al., 2016).

#### 4.2.4 Poly (ADP-Ribose) Polymerase Inhibitors-Immunotherapy Combination

Jiao et al. have concluded that PD-L1 expression was upregulated by PARP inhibitor in cell lines of breast cancer by inactivated GSK3, resulting in impaired anticancer immunity. In addition, the anti-PD-L1 therapy and PARP inhibitor combination show higher therapeutic effectiveness (Jiao et al., 2017). Independent of BRCA1/2 mutation, PARP inhibitor-mediated immune response modulation accords to the therapeutic value. According to recent findings, PARP inhibitors promote the buildup of cytosolic fragments of DNA as a result of unsolved DNA lesions, further activate the DNA-sensing cGAS-STING pathway which results in stimulating type I interferon production to further stimulate anti-tumor immunity which is independent of BRCA status (Shen et al., 2019).

#### 4.2.5 Poly (ADP-Ribose) Polymerase Inhibitors-Epigenetic Drugs Combination

As stated previously, epigenetic modifications were linked to PARP inhibitor sensitivity (Kondrashova et al., 2018; Guo et al., 2018; Fukumoto et al., 2019). The two notable mechanisms of post-translational gene expression regulation are histone acetylation and deacetylation (Audia and Campbell, 2016). Studies concluded that treatment of cancer cells with histone deacetylation (HDAC) inhibitor and PARP inhibitor had a synergistic effect because of the induction of HDAC inhibitor on homologous recombination deficiencies, which further sensitized the cancer cells to PARP inhibitor (Jasek et al., 2014; Baldan et al., 2015; Ha et al., 2014; Min et al., 2015). Numerous mechanisms have been discovered. Initially, it was found that HDAC inhibitor reduced the DNA repair gene expression of BRCA1, RAD21, CHK1, and RAD51 via the transcription factor E2F1 (Kachhap et al., 2010). Additionally, HDAC inhibitors inhibited HSP90 deacetylation, leading to the degradation of its substrate RAD52, CHK1, BRCA1, and ATR (Kim et al., 2017). Eventually, recent research has elucidated that acetylation inhibited DNA damage-induced chromatin PARylation and that HDAC inhibitor therapy significantly enhances PARP1 trapping at DSB sites in chromatin (Robert et al., 2016; Abbotts et al., 2019). Besides that, the DNA methyltransferase (DNMT) inhibitors in low doses stimulated BRCAnes by decreased regulation of key homologous recombination gene expression. The DNMTi and PARP inhibitor combination increased cytotoxicity by enhancing the PARP “trapping” upon double-stranded break sites independent of BRCA mutation (Pulliam et al., 2018; Muvarak et al., 2016). However, the data to evaluate the clinical effectiveness remain unavailable and need further research.

## 5 Conclusion

Since TMZ is the first-line drug that is effective in the treatment of GBM, resistance to it is the most serious problem in the context. PARP inhibitors have recently been used to overcome TMZ resistance. However, there are a number of factors that make PARP inhibitors' clinical development challenging. The limited BBB penetration, resistance to PARP inhibitors, and hematological toxicities that occur when PARP inhibitors are used in combination with TMZ/IR are three of the most significant challenges in PARP inhibitors’ clinical development. As a result, this review concludes that challenges can be overcome if good strategies are implemented. For example, if targeted delivery is used, the drug can penetrate the BBB and act. The prevention of off-target toxicity is the second advantage of targeted delivery. Third, we can overcome PARPI resistance by using combination therapies. The use of targeted delivery and combination therapies is one step toward achieving the goal of successful clinical development of PARP inhibitors in GBM.
